# New Horizons: COVID-19 and the Burden of Neuropsychiatric Illness in Pakistan

**DOI:** 10.12669/pjms.36.COVID19-S4.2792

**Published:** 2020-05

**Authors:** Ali M. Hashmi, Haider Ali Saleem

**Affiliations:** 1 Ali M. Hashmi, MBBS, MD, DABPN, FAPA Associate Professor of Psychiatry, King Edward Medical University/Mayo Hospital, Lahore, Pakistan; 2 Haider Ali Saleem, MBBS. Aga Khan University, Karachi, Pakistan

**Keywords:** Mental illness, COVID-19, mental health, Healthcare workers

## Abstract

This manuscript reviews the current state of knowledge about the burden of mental illness and assesses the impact of COVID-19 illness on mental health in Pakistan. For this we analyzed secondary data obtained from the Institute of Health Metrics and Evaluation. The Global Burden of Disease (GBD) study draws from a wide range of data sources to quantify global and regional effects of a disease. We also did a literature search on the effects of COVID-19 illness on mental health and the psychosocial effects of COVID-19 and other Corona virus related illnesses such as SARS-CoV and MERS-CoV. Data from the studies obtained was utilized to extrapolate the anticipated effects of COVID-19 illness on healthcare workers, COVID-19 patients and the general public in Pakistan. Mental illness poses a significant challenge to Pakistan’s under resourced health care system. COVID-19 has the potential to strain Pakistan’s healthcare system to the breaking point. So far, the general morbidity from COVID-19 illness in Pakistan has been low compared to other countries but this could change in the coming weeks and months. Hidden within this crisis are also some opportunities for both healthcare and education.

## INTRODUCTION

Neuropsychiatric illnesses pose an enormous burden both socially and economically in Pakistan. The prevalence of common mental illnesses is high and resources to deal with these illnesses are limited leading to considerable morbidity. Lack of adequate mental health services imposes a significant public health burden since untreated mental illnesses contribute both directly and indirectly to morbidity and mortality from physical illnesses. COVID-19 has posed a tremendous challenge to Pakistan’s already strained healthcare system particularly with regards to mental health and psychosocial support services. COVID-19 illness has the potential to further impact Pakistan’s already overburdened health system but is also accelerating some positive changes both in healthcare delivery and education.

Pakistan is the world’s fifth most populous country with an estimated population of 220 million as of 2020.^1^ Neuropsychiatric illnesses, including mental illness pose a huge economic burden on society globally.^2^ With an increase in societal awareness and much needed de-stigmatization in recent years, increasing numbers of people are seeking help for common mental illnesses and the few studies that have been done in Pakistan have documented a much higher prevalence of mental health issues in Pakistan compared to Western countries. One systematic review estimated the mean prevalence of anxiety and depressive disorders in the community to be 34% (29%-66% for women, 10%-33% for men).^3^ There are an estimated six million drug addicts in the country. Serious mental illnesses such as schizophrenia are estimated to have a prevalence rate of 1.5% in the population.^4^ Child mental health problems also are common with a mean prevalence of 15%.^5^

### The Economic Burden of Mental Illness in Pakistan

While local data are scarce, one study estimated that the cost of mental illness to society in Pakistan was PKR 250,483 million (USD 4,264 million) in 2006^6^, with direct costs in the form of medical care accounting for 37%, with the rest being indirect costs. These costs have, undoubtedly, grown exponentially larger since 2006. Importantly this burden falls entirely on individuals who have to make out of pocket payments for all health care, as the country has no national health insurance plan.

### Why Mental Health? The link between Mental and Physical Health

Increasing evidence points to the importance of mental health as a critical measure of overall health with more and more research emerging to show how mental and physical health are linked. Multiple studies have documented how untreated mental illness causes significantly worse outcomes in medical illness and conversely, how various medical illnesses can be a risk factor for, and worsen the prognosis for, mental illness e.g. depression can worsen the health outcomes of other chronic illnesses, such as angina, asthma, diabetes, and arthritis.^7^ The risk of dying from an initial myocardial infarction is higher in patients with depression and varies with the severity of the depressive episode, and clinically severe depressive symptoms significantly increase the risk of death from cardiovascular disease and stroke.^8^ In bipolar disorder too, there exists a higher risk of mortality from both cardiovascular and respiratory diseases.^9^ Patients with anxiety disorders often have multiple medical co-morbidities including chronic pain, gastrointestinal, respiratory, cardiovascular and endocrine illnesses.^10^ Individuals with schizophrenia and other severe mental illnesses die at a younger age than the general population and this excess mortality results in part from a higher prevalence and greater severity of multiple co-morbid medical conditions including diabetes, respiratory illness, and cardiovascular disease.^11^ Neuropsychiatric and medical illnesses can thus no longer be viewed in isolation. Effective treatment of medical illnesses requires that equal attention be paid to neuropsychiatric (including mental) illness to reduce the functional burden of disease and prevent excess morbidity and mortality from medical illness.

### Pakistan’s Mental Health Gaps

Despite the massive economic burden of mental diseases and the clear connections between mental and psychological illnesses, mental health has always been a low priority for Pakistan’s governments. Pakistan’s public health budget is less than one percent of the country’s GDP and mental health does not even have a separate budget. The World Health Organization-Assessment Instrument for Mental Health Systems (WHO_AIMS) report on the mental health system in Pakistan published in 2009^12^ reported alarming deficiencies in financial, human and logistical resources. Only 0.4% of all health care expenditure by the government, estimated to be US$ 82,304.24, was allocated for mental health, with 3729 outpatient mental health facilities, only five mental health hospitals, a total of 5,056 beds (2.7 beds per 100,00 population), and 342 psychiatrists (0.2 per 100,000 population). No significant amendments in government policy addressing mental health have been made since then, while the burden of neuropsychiatric illness has continued to grow across all age groups and genders.

Decades of political and social instability, terrorism, natural disasters, inflation and unemployment are just some of the many risk factors that contribute to the increasing prevalence of mental disorders in Pakistan. With an ever deteriorating socioeconomic situation, Pakistan’s public health system stood at a breaking point in mental health even before the COVID-19 pandemic, perhaps the most serious global public health emergency in the last 100 years.

### COVID-19 and the crisis of Mental Health in Pakistan

The WHO declared COVID-19 a pandemic on 11^th^ March 2020. Since then the number of cases worldwide have grown to 3,588,773 with two lac forty seven thousand five hundred three (2,47,503) deaths (as of 6^th^ May 2020)^13^. Low income countries are particularly susceptible due to a severe shortage of healthcare resources. As of May 6^th^, 2020, Pakistan has reported 22,533 positive cases with about five hundred twenty six deaths^13^ with experts predicting that the worst is yet to come. The country remains in a state of partial lockdown.

Data about this novel virus continues to evolve at a rapid pace but similar outbreaks involving viruses from the same family such as SARS-CoV in 2003 and MERS CoV in 2012^14^ offer some guidance. Both these outbreaks were also caused by viruses belonging to the corona virus family and featured high infectivity. By reviewing their impact on the psychological and emotional health of people, we can reasonably predict the psychological effects of COVID-19 illness.

**Who will be affected?**

The psychological effects on any community affected by COVID-19 illness can be broadly divided into three categories: the uninfected public, patients and healthcare workers (HCWs).

In the public (uninfected people), a general sense of fear and uncertainty prevails (of being infected, of family members especially children, the elderly or the medically ill getting sick or dying), worsened by the constant media reports of new cases including deaths. During the SARS outbreak, studies reported a greater sense of personal danger due to constant media coverage of the spreading disease.^15^ A survey of over 1200 people in China reported that during the initial phase of the current outbreak, half the respondents rated the psychological impact of the pandemic at moderate to severe and about one third reported moderate to severe anxiety.^16^ While data specific for Pakistan does not yet exist, it is safe to say that the psychological burden will be significant due to a multitude of reasons: overcrowded and under resourced hospitals may get overwhelmed if cases spike; low literacy rates mean people have an incomplete understanding of the situation and may continue with ‘life as usual’ despite warnings by healthcare workers and the government and an economic downturn in a country where millions depend on daily wages may leave many with no income at all for extended periods of time.

Patients with the illness also undergo significant mental trauma and stress. Being infected with a viral illness with no approved ‘cure’, poorly understood modes of transmission and no lab tests to monitor daily progress causes a lot of anxiety for the patients.^17^ A study during the SARS outbreak showed that apart from fear of the disease itself and its prognosis, patients were worried about being stigmatized, about the wellbeing of family and friends and about lost income.^18^ In quarantine centers, spikes of anxiety were seen in patients correlating with spikes in their fevers and feelings of loneliness and helplessness were prevalent in patients. In Pakistan, there have been multiple reports of patients running away from treatment centers and many refusing to seek medical help due to a fear of being quarantined.^19^

The outbreak also has a severe psychological impact on the HCWs. HCWs, especially the ones caring for COVID 19 patients, report high levels of stress all across the world. Shortage of supplies including required medicines, personal protective equipment (PPE) and inadequate support are some factors contributing to the psychological burden of health care professionals.^20^ They report symptoms of anxiety, depression, psychological distress and insomnia. During the SARS outbreak, HCWs reported exhaustion due to hours spent putting on and taking off protective equipment in the day and the greatest fear reported was that of infecting a loved one at home after duty hours.^15^ HCWs also witness the trauma of colleagues contracting the virus, being intubated and switching roles from health provider to patient and many losing their lives in the process. HCWs also have a higher chance of experiencing “moral injury” as they put their lives on the line for professional duty amidst a dire shortage of appropriate resources.^21^ An upcoming study from Lahore (the first in Pakistan) by Nazish Imran and colleagues documents the high incidence of anxiety, depression and insomnia in HCWs caring for COVID-19 patients.

### Silver Linings

Despite the dire situation around the world and in Pakistan, all is not doom and gloom. Pakistan’s current case fatality ratio from COVID-19 illness is relatively low compared to many Western countries including the UK, USA and Italy.^22^ The reasons for this are unclear at the moment but may include lower number of tests being performed and statistical under reporting of all-cause mortality as well as other, yet to be investigated factors including difference in virus strains, pre-existing cross immunity in the population etc. The fact remains though that Pakistan’s poorly resourced healthcare system has so far been able to manage the rising cases of COVID-19 in adequate fashion, no doubt partly due to the dedication and hard work of its HCWs.

In addition, the unprecedented global lock down due to COVID-19 has hastened the adoption of computer technology by both healthcare institution as well as institutions of higher learning with Tele-Health centers blossoming all across the country.^23^ This will, in the longer term, enable quality healthcare to reach hither to underserved peri-urban and rural areas to provide higher quality healthcare to these areas.

Pakistan’s Higher Education Commission, the regulatory body for all higher education in the country has also mandated that all institutions of higher learning (including medical colleges and universities) move to ‘virtual/online’ teaching and assessment with immediate effect.24 While this may involve some initial obstacles, in the medium to long term, it will enable a much broader reach of institutions of higher education to segments of Pakistan’s population that were deprived of quality education. In addition, the move to virtual/online teaching, training and healthcare service and delivery will enable easier links with international educational institutions for much needed technology and expertise transfer to Pakistan.

## CONCLUSIONS

COVID-19 continued to pose an unprecedented challenge to healthcare workers and to humanity at large. Pakistan, with an already severely under-resourced health care system, is particularly vulnerable to these challenges. So far, Pakistan’s public healthcare system, despite longstanding structural challenges has responded admirably to the threat of COVID-19 illness. In addition, COVID-19 illness has accelerated some much needed changes in Pakistan’s healthcare and education systems that, if sustained, have the potential to transform these crucial social sectors for the benefit of the most deprived segments of Pakistan’s population and take the country forward into the twenty first century.

### Authors’ Contributions

**AMH:** General supervision of the research group, acquisition of patient data, interpretation of data, critical appraisal of data, write-up of manuscript, final approval of version to be published.

**HAS:** Acquisition of patient data, analysis, interpretation of the data, drafting of the article, write up and revision of the manuscript and final approval of version to be published.
